# The effects of *Saccharomyces boulardii* on rat colonic hypermotility induced by repeated water avoidance stress and the potential mechanism

**DOI:** 10.7717/peerj.14390

**Published:** 2022-11-22

**Authors:** Jingwen Liu, Haixia Ren, Fangting Yuan, Ming Shao, Hesheng Luo

**Affiliations:** Department of Gastroenterology, Renmin Hospital of Wuhan University, Wuhan, Hubei Province, P. R. C

**Keywords:** Gastrointestinal motility, Chronic stress, *Saccharomyces boulardii*, TLR4, Gut microbiota, Irritable bowel syndrome

## Abstract

**Background:**

*Saccharomyces boulardii* (*Sb*) has been reported to have the potential to regulate gut motility. The aim of this experiment was to explore the possible function of *Sb* in gut hypermotility elicited by repeated water avoidance stress (WAS).

**Methods:**

Adult male Wistar rats (*N* = 24) were divided into one of the following three groups: control (C), NS (normal saline) + WAS group (N), and *Sb* + WAS group (S). A diarrhea-predominant irritable bowel syndrome (IBS-D) model in rats was induced using the WAS method. Gut motility was evaluated by stool pellet expulsion per hour. The contractile activity of the colonic muscle strips was measured using an RM6240 multichannel physiological signal instrument. qRT-PCR and immunohistochemistry were used to assess Toll-like receptor 4 (TLR4) expression in colon tissue. ELISA was used to measure the level of cytokines in the serum and colonic tissue. Also, the microbiota composition was determined using high-throughput 16S rRNA sequencing.

**Result:**

The results showed that oral *Sb* decreased the WAS-induced increased defecation and colonic hypermotility *in vivo*. Furthermore, *Sb* also decreased the contractile amplitude of colonic circular muscle (CM) and longitudinal muscle (LM) strips in a dose-dependent manner *in vitro*. Repeated WAS increased TLR4 expression, but *Sb* reversed it. *Sb* also reduced interleukin-6 (IL-6), IL-1β, and interferon-γ (IFN-γ) levels in serum and colonic tissue, while increasing IL-10 levels in colonic tissue. Meanwhile, the rats from the NS + WAS group had decreased microbiota diversity and had lower relative abundances of Patescibacteria, Epsilonbacteraeota, Cyanobacteria, and Turicibacter compared with controls. The rats in the *Sb* + WAS group showed a tendency to increase the relative abundance of Blautia when compared to control rats and had lower relative abundances of Acidobacteria and Anaerostipes compared with the NS + WAS group.

**Conclusion:**

Our findings demonstrated that *Sb* improved colonic hypermotility in rats, reversed the high-expression of TLR4 in the colon caused by repeated WAS, modulated cytokines in the colon and serum, and altered the gut microbiota, indicating that *Sb* may be useful for IBS-D.

## Introduction

IBS is a commonly occurring non-organic gastrointestinal disorder associated with chronic abdominal discomfort and bowel habit change  (Enck et al., 2016). Up to now, the mechanisms of IBS are not yet fully understood. Altered visceroception and gut motility have been reported as key pathophysiological mechanisms in IBS (Rychter et al., 2014; Liang et al., 2012). IBS is strongly correlated with a number of psychiatric diseases, such as depression and dysphoria, and stress is also highly linked with IBS (Chang, 2011; Qin et al., 2014). Substantial research has revealed that chronic stress increases gut motility and transit (Taguchi et al., 2017; Subramaniyam et al., 2020). When the natural rhythm of intestinal contraction is altered, it may cause common hypomotility or hypermotility disorders, such as IBS (Ma et al., 2018; Beyder & Farrugia, 2012). Rats treated to repeated WAS, a chronic stress intervention, are now commonly utilized in IBS animal models (Bradesi et al., 2005). Repeated WAS-loaded rats typically exhibited increased defecation and enhanced colonic motility, mimicking the human IBS-D condition (Yuan et al., 2022). Chronic stress also causes an immune system response (Gao et al., 2018). Toll-like receptors (TLRs) are members of the transmembrane pattern-recognition receptor family and serve as a bridge between innate immunity and acquired immunity. TLR4 was one of the first members of the transmembrane pattern-recognition receptor family to be studied. At present, studies have demonstrated that TLR4 is up-regulated in IBS-D patients and in repeated WAS-induced rat models (Arie et al., 2019; Jalanka et al., 2021).

The gut microbiota has a significant impact on intestinal homeostasis  (Artis, 2008). Changes in the gut microbiome may be linked with bowel disorders. Multiple studies have shown that intestinal flora exerts an important role in the pathogenesis of IBS (Simren et al., 2013). Several genomic investigations have elucidated the significant alterations in the intestinal flora of IBS patients and the function of intestinal flora imbalance in the etiopathogenesis of IBS (Mars et al., 2020). At certain doses, probiotics are microorganisms that assist the host body in staying healthy. Many probiotics are derived from the gastrointestinal tract and are associated with the intestinal microbiota (Hill et al., 2014). Probiotics have an immunomodulatory value and are crucial in both the protection of the intestinal mucosa and the secretion of antibacterial substances (Ciorba, 2012; Wan, Forsythe & El-Nezami, 2019). In several studies, probiotics have been shown to influence the host’s gut microbial composition and immunological responses (Vieira, Teixeira & Martins, 2013). In parallel, given their impressive safety profile, the use of probiotics in the therapy of IBS has become increasingly popular (Suez et al., 2019).

*Sb* is a yeast strain that has been extensively examined for its probiotic properties. Studies have revealed that Sb is resistant to gastric acid, generally grows well at 37 °C, and suppresses a variety of pathogenic bacteria growth both *in vivo* and *in vitro* (Pais et al., 2020; Czerucka, Piche & Rampal, 2007). Meanwhile, *Sb* has been shown to be useful in the therapy of a multitude of diseases, such as inflammatory bowel diseases (IBD) and various forms of diarrhoea (Szajewska & Kolodziej, 2015). According to several findings, *Sb* boosts the intestinal immune response and strengthens the intestinal barrier (Vieira, Teixeira & Martins, 2013). Previous studies have shown that *Sb* suppresses gut motility in the IBS-D mouse model, and clinical trials have confirmed the benefit of *Sb* in IBS patients (McFarland, 2010; Gu et al., 2022). However, the deep-level relationship between *Sb* and chronic stress-induced IBS remains largely unclear.

We investigated the regulatory effects of *Sb* on chronic stress-induced colonic dysmotility in IBS-D rats, as well as the expression levels of TLR4 in colonic tissues, cytokine levels in serum and colonic tissues, and changes in gut microbiota, and determined the potential role of *Sb* in a chronic stress-loaded rat model of IBS-D.

## Materials & Methods

### Animals

The male Wistar rats (200–220 g) with a certified report were acquired from Vital River Laboratories (Beijing, China) and bred in a constant temperature (20 °C∼25 °C) and humidity (50%∼65%) environmentally controlled chamber with a 12-hour light/dark cycle. Rats received sterile food and water freely. Padding was changed twice a week. Rats were euthanized by inhalation of CO_2_ at the end of the experiment. Animal ethics approval was granted by the Laboratory Animal Welfare and Ethics Committee of Renmin Hospital of Wuhan University (approval ID: NO.20210308) and all procedures were in accordance with the Experimental Animal Protection Committee of the Ministry of Health of the People’s Republic of China.

### Repeated WAS protocol

We placed the rats on a circular platform (9 × 9 × 15 cm) fastened to the center of a tank (40 × 40 × 50 cm) containing water for one hour per day for ten days, according to prior test techniques in our laboratory. The amount of water in the tank was kept at a maximum height of one centimeter below the platform. The controls were put on the same platform as the experimental animals, but were not given any water. That is, the control rats received sham water avoidance stress (SWAS). The above experiment was implemented at 10:00 am each day. During the whole experiment, the defecation of each rat was recorded for subsequent statistical analysis.

### Experimental design

The rats were randomly divided into three groups after three days of adaptive feeding (*n* = 8 per group): (i) control; (ii) NS + WAS; and (iii) *Sb* + WAS. *Sb* was purchased from Biocodex (Gentilly, France) and contained ≥1.3 × 10^9^ CFU/g viable *Sb*. Rats in the *Sb* + WAS group were gavaged for 14 consecutive days with *Sb* saline suspension (0.5 g/kg/d). The dose of *Sb* was defined based on the dose administered to rodents in previous studies. The NS+ WAS rats were given the same volume of NS via gavage. Only distilled water was given to the control rats. From the 15th day, the rats in the NS+WAS group and the *Sb* + WAS group were treated with WAS for 1 h after the conventional gavage for 2 h for 10 consecutive days, and the rats in the control group received SWAS. All rats were euthanized on day 25, and their blood was collected to obtain serum and stored at −20 °C for future analyses. The colon was removed aseptically to measure motility. Stool samples were also collected and set aside at −80 °C.

### H&E staining

Rats were euthanized, and the excised proximal colon tissue was immersed in 4% paraformaldehyde and fixed at 4 °C for 24 h. Following that, samples were dehydrated in various concentrations of ethanol before being paraffin embedded. After that, the embedded paraffin samples were cut to a thickness of 4 µm. Subsequently, the sections were stained at room temperature with hematoxylin and eosin (H&E). The stained sections were scanned using NDP.view 2 software, and the structure, length, and integrity of colonic villi were observed.

### Colonic motility tests

The proximal colons of rats were carefully removed, the intestinal lumen was opened along the mesenteric border, and the intestinal contents were then cleaned with cold Ca2 +-free saline solution perfused with 95% O_2_ and 5% CO_2_, sheared along the vertical and horizontal directions of the long axis of the colon to prepare CM and LM strips (8 mm long and 2 mm wide), respectively. We immersed the prepared smooth muscle (SM) strips in a thermostatic bath with 5 mL of Tyrode buffer containing a mixture of 95% O2 and 5% CO2. The ends of the colonic SM strips were fastened to the thermostatic bath containing the Tyrode’s buffer and to the isometric force transducer (JZJOIH, Chengdu, China). SM strips were incubated under initial resting tension (1.0 g for CM strips and 1.5 g for LM strips) until the amplitude was at equilibrium. The contraction of the CM/LM strips in each group of rat colon was measured using the RM6240 multichannel physiological signal system (Chengdu, China). In addition, SM strips were prepared from the isolated proximal colons of rats in the model group. Similarly, changes in colonic SM tone were measured with a tension transducer. After a period of regular contraction signals, incubation of colonic SM strips by adding a concentration gradient of *Sb* solutions to the thermostatic bath, we determined the *in vitro* administration concentration of *Sb* by pre-experiment (final incubation concentrations of CM strips were 1, 2, and 3 mg/mL, respectively; final incubation concentrations of LM strips were 0.5, 1, and 1.5 mg/mL, respectively). The changes in spontaneous contractile activity were observed and recorded.

### Immunohistochemistry

The paraffin sections of the colon were dewaxed using an environmentally friendly dewaxing agent and then dehydrated in graded alcohol (100%, 95%, and 75%) for 5 min each. Antigen repair, blocking, and other steps were carried out in strict accordance with the kit instructions. Sections were incubated with rabbit anti-TLR4 antibody (1:200, bs-23927R, Bioss) overnight at 4 °C and with secondary antibody for 45 min at 37 °C. Finally, freshly prepared DAB working solution was added to the tissue sections and observed under the microscope, with the color development terminated by tap water. The sections were then re-stained with hematoxylin, dehydrated and dried in gradient alcohol, and sealed with an environmentally friendly sealant. The sections were scanned using NDP.view 2 software and a specialist pathologist evaluated the images and calculated HSCORES without knowing the grouping, where HSCORE = ΣPi(i +1), (Pi: The number of positive cells as a percentage of the number of all cells in the section; i: Coloring intensity).

### q RT-PCR

Total RNA was extracted from rat colon using Trizol reagent (Takara, Japan), and cDNA was obtained with a reverse transcription reagent kit (Takara, Japan). The LightCycler 480 SYBR Green I Master (Roche, Basel, Switzerland) and the LightCycler 480 qPCR device were used for the qPCR. The relative expression level of TLR4 was calculated using 2^−ΔΔCT^ method. The primer sequences used for amplification were as follows: the upstream primer for TLR4 is 5′-TCCAGAGCCGTTGGTGTATC-3′, the downstream primer is 5′-GAGCATTGTTCCTCCCACTCG-3′; the upstream primer for GAPDH is 5′-GACATGCCGCCTGGAGAAAC-3′, the downstream primer is 5′-AGCCCAGGATGCCCTTTAGT-3′.

### ELISA

ELISA assay kits (MULTI SCIENCES, Hangzhou, China) were used to measure IL-6, IL-1β, IL-10, and IFN-γ in serum and colonic tissue homogenates. The experimental steps were in strict accordance with the manufacturer’s instructions.

### 16S rRNA Gene Sequencing

Extraction of DNA from stool samples via the Mag-Bind® Stool DNA Kit (Omega, Guangzhou, China) as per the kit’s instructions. Electrophoretic separation was performed with a 1.2% agarose gel, and the purity and concentration of the isolated DNA were determined using a NanoDrop NC2000 spectrophotometer (Thermo Science, Waltham, USA). The primers 338F (5′-ACTCCTACGGGAGGCAGCA-3′) and 806R (5′-GGACTACHVGGGTWTCTAAT-3′) were utilized to amplify the hypervariable V3 and V4 regions of the bacterial 16S rRNA gene. PCR amplification was performed with Q5® High-Fidelity DNA Polymerase from New England Biolabs. The amplification cycle was controlled to minimize the number of amplification cycles and to ensure identical amplification conditions for the same batch of samples. PCR amplification and purification of amplification products were used for library preparation and pyrosequencing, which were performed at paired-end 250 bp on the Illumina NovaSeq platform (Illumina, San Diego, USA) by Personal Biotechnology, Co., Ltd. (Shanghai, China).

### Statistical analysis

The data from this experiment was statistically analyzed using SPSS Statistics 28.0 (IBM SPSS, Chicago, IL). All results were expressed as mean ± SEM. Significant differences between all groups were assessed with the use of the independent sample *t*-test, one-way ANOVA test with subsequent Tukey’s multiple comparisons test, or Kruskal-Wallis test, Bonferroni correction was applied to multiple testing. *P* < 0.05 indicated the presence of statistical significance.

## Results

### Histopathological observation

**Figure 1:**
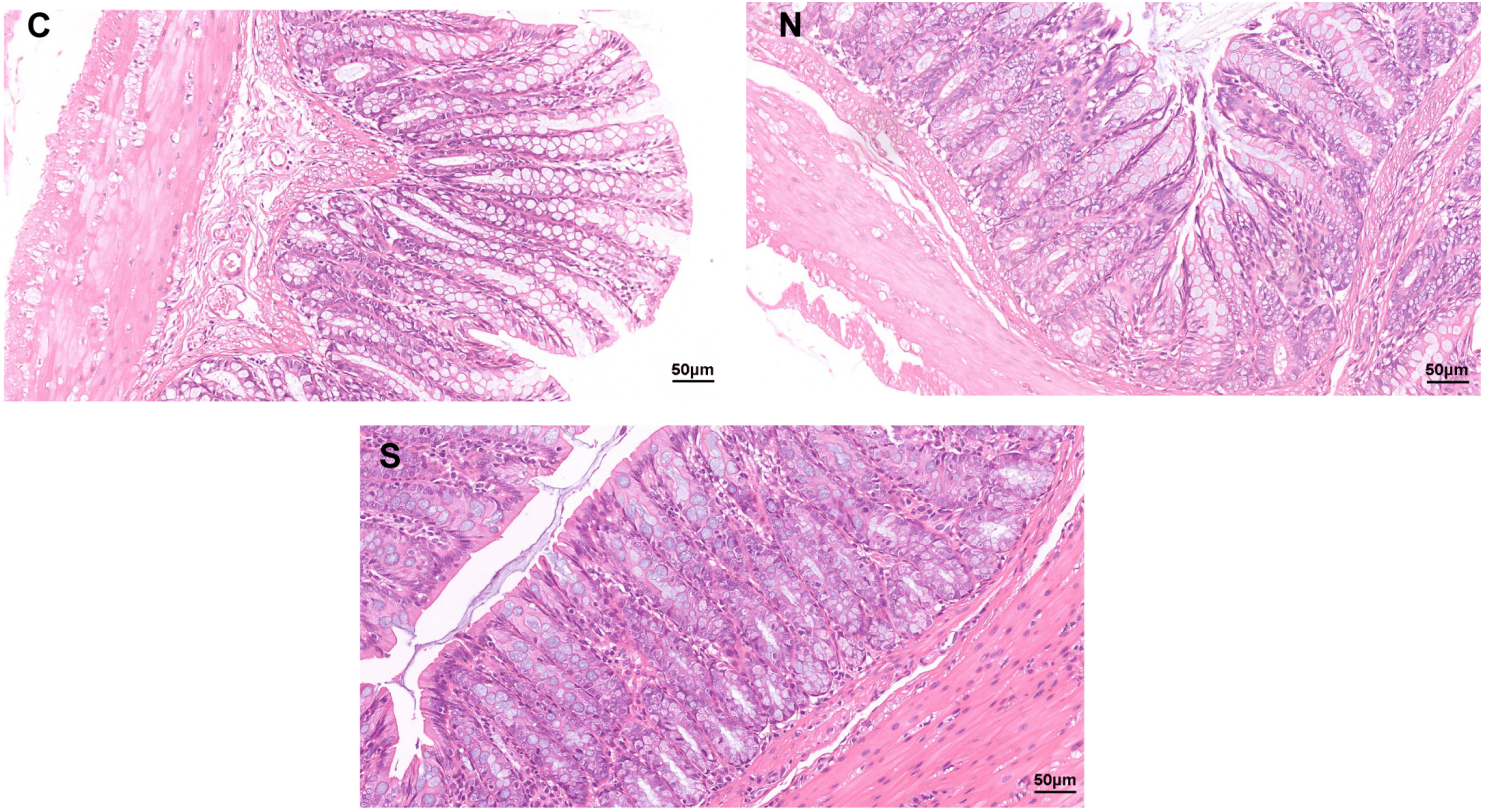
Pathological changes of colon tissue in rats (H&E staining, ×400). C, control group; N, NS+WAS group; and S, Sb +WAS group. (*n* = 8 per group).

As shown in Fig. 1, no clear pathological changes were observed in the H&E staining of the colonic tissues of rats in each group. The colon structure of all three groups was clear, no inflammatory cells infiltrated or tissue damage was evident in the colonic mucosa of any of the groups. The mucosal epithelium appeared intact, the arrangement of intestinal glands was in good condition, and goblet cells were abundant. However, repeated WAS caused mild villi damage, and the microstructure of the colonic mucosa was similar in the WAS +* Sb* group and the control rats. Fig. 1 shows longer villi length in the WAS + *Sb* rat and shorter villi length in the WAS+NS rat. The observations outlined above suggest that oral administration of *Sb* could decrease the damage of colonic villi in IBS-D rats.

### The fecal pellets expulsion

**Figure 2:**
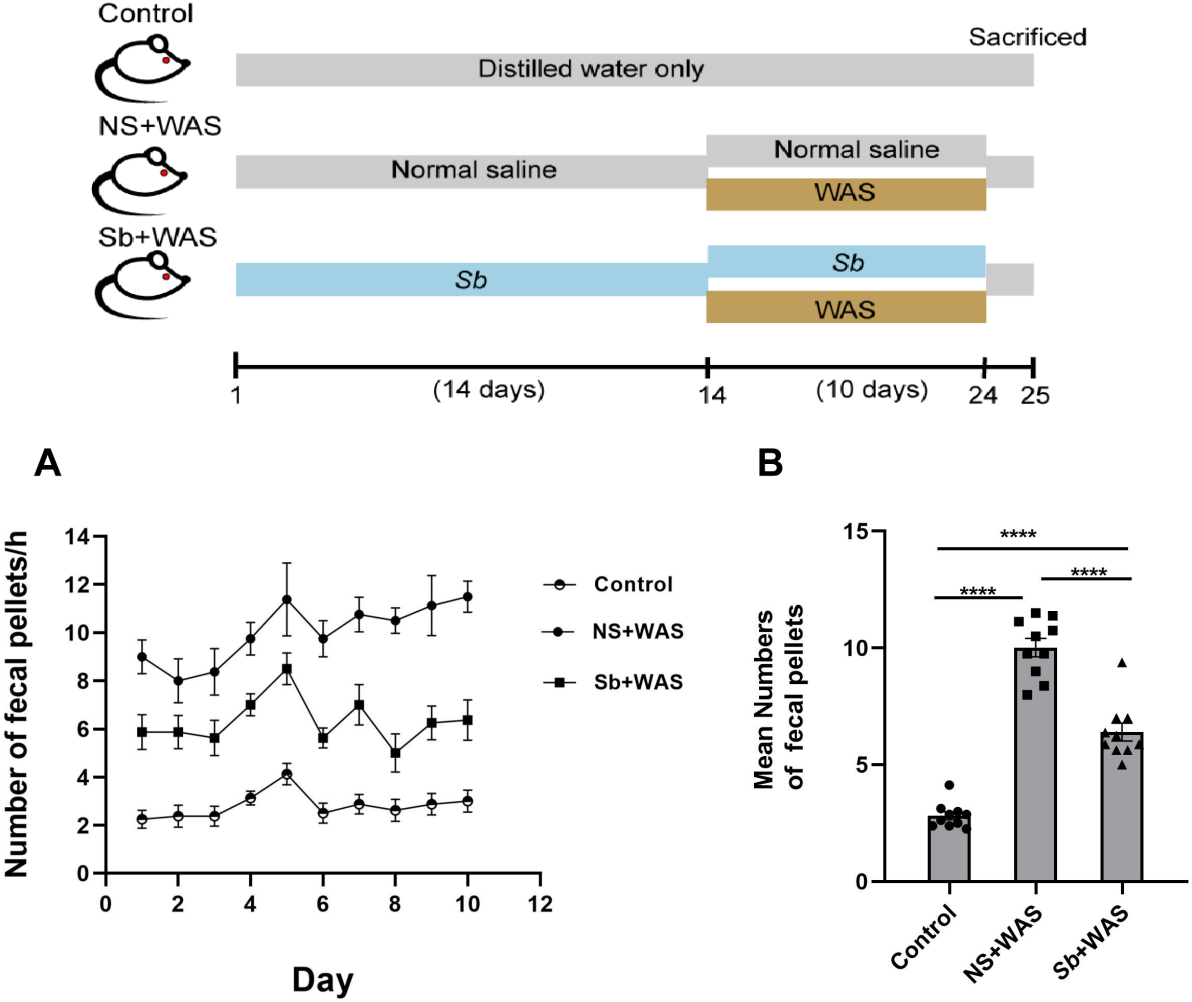
Rats were continuously performed with repeated WAS for 10 days with or without *Sb* pretreatment for 14 days. (A) A line graph of the defecation of three groups of rats over 10 days. (B) A bar graph depicting the average number of fecal expulsions in three groups. (*n* = 8 per group, **** *P* < 0.0001).

Chronic stress enhanced the motility of the colon significantly. Here, we assessed the colonic motor function of rats using the quantity of fecal pellets defecated during a 1-h period. Figure 2A represents the fecal pellet excreted by the rats from the control, NS + WAS, and *Sb* + WAS groups during the session. Figure 2B shows the average number of fecal pellets excreted by the three groups of rats during the 10-day WAS experiment. Repeated WAS accelerated **fecal pellet** **expulsion**. Rats in the NS+WAS group excreted more fecal pellets than controls (NS + WAS 10.01 ± 0.39 vs Control 2.82 ± 0.17, *P* < 0.0001). Rats in the *Sb* +WAS group defecated less than rats in the NS+ WAS group (*Sb* + WAS 6.40 ± 0.38 vs NS + WAS 10.01 ± 0.39, *P* < 0.0001), although it was still greater than controls (*Sb* + WAS 6.40 ± 0.38 vs Control 2.82 ± 0.17, *P* < 0.0001).

### The effects of repeated WAS on colonic strip contractile function

**Figure 3:**
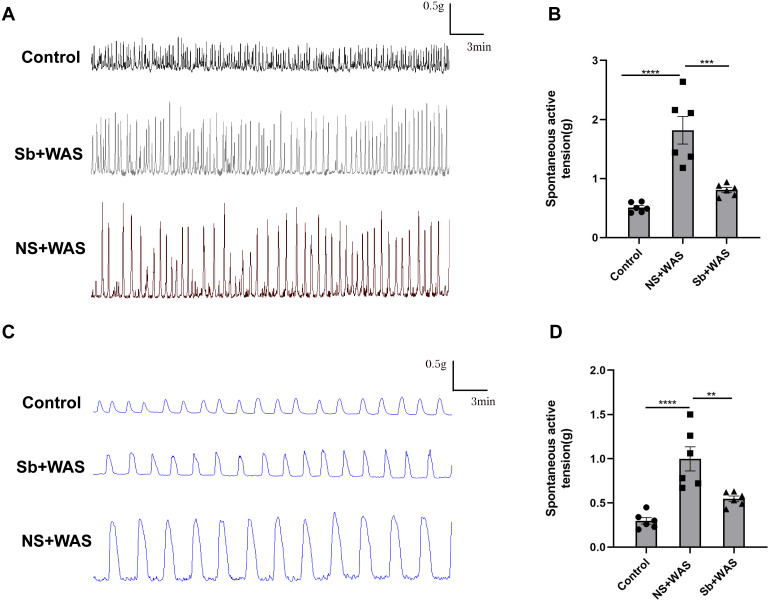
Effects of repeated WAS on spontaneous contraction of proximal colonic muscle strips. (A) Representative images of spontaneous contractions of CM *in vitro* in NS + WAS, *Sb* + WAS, and control rats. (B) The average amplitudes of the CM contractile activities of three groups. (C) Representative images of spontaneous contractions of LM *in vitro* in NS + WAS, *Sb* + WAS, and control rats. (D) The average amplitudes of the LM contractile activities of three groups. (*n* = 6 per group. ** *P* < 0.01; *** *P* < 0.001; **** *P* < 0.0001).

In comparison to the control rats, repeated WAS dramatically enhanced spontaneous contraction of the isolated proximal colon. The mean contraction amplitude of CM strips in NS+WAS rats was statistically upregulated compared to controls (1.82 ± 0.23 g vs 0.53 ± 0.03 g, *P* < 0.0001). A similar phenomenon has been observed in Fig. 3D. The LM strips contraction amplitude was obviously higher in NS + WAS rats than in controls (1.00 ± 0.01 g vs 0.30 ± 0.04 g, *P* < 0.0001). However, oral administration of *Sb* reversed the enhanced contraction of the proximal colon in rats caused by repeated WAS *in vivo*.

### *Sb* alleviate colonic hypermotility *in vitro*

**Figure 4:**
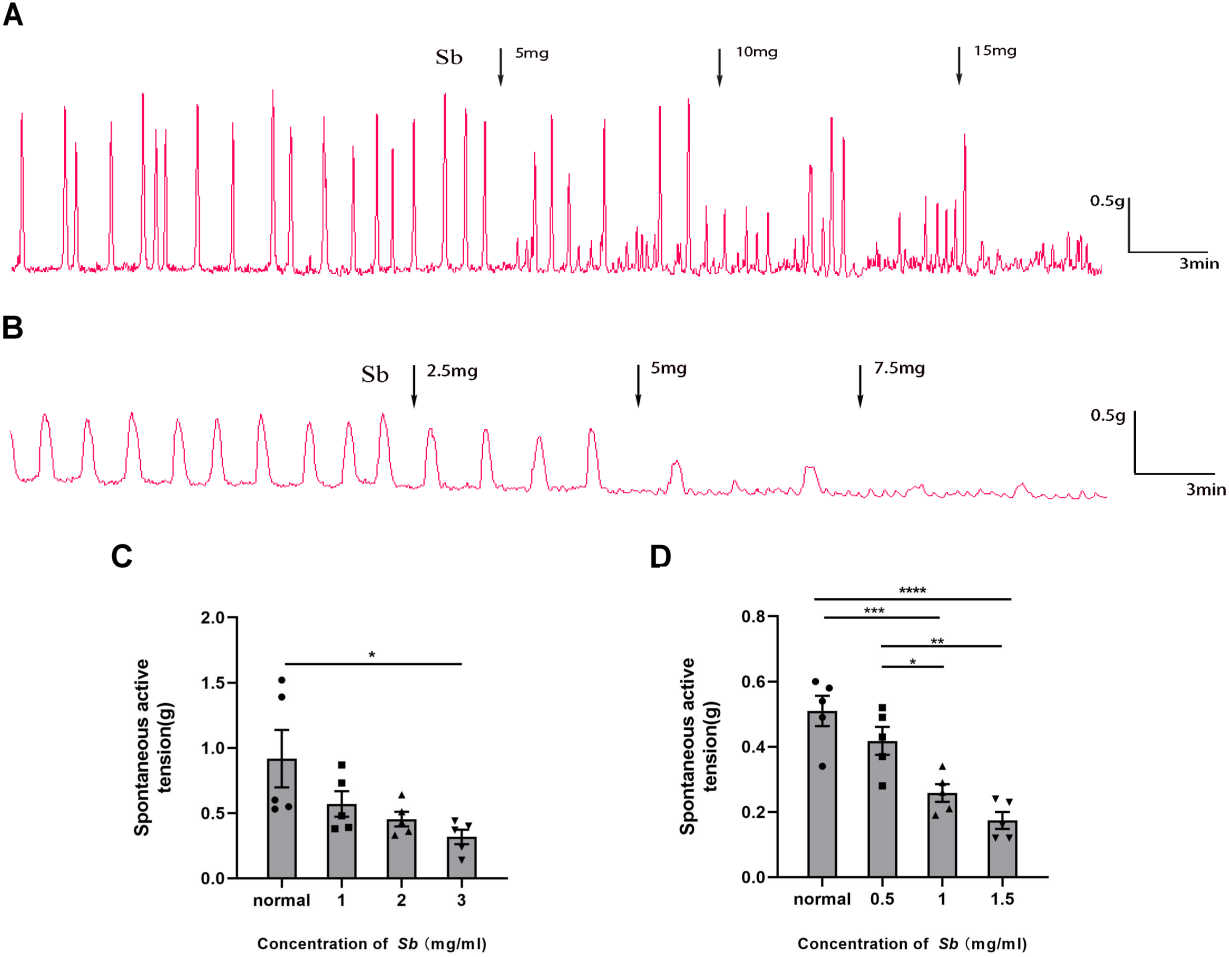
Effect of *Sb* on spontaneous contraction of colonic muscle strips. (A) Effects of *Sb* on spontaneous contraction of CM strips. (B) Effects of *Sb* on spontaneous contraction of LM strips. (C) Bar graphs reflecting the results of CM contractile amplitude. (D) Bar graphs reflecting the results of LM contractile amplitude. (*n* = 5 per group. * *P* < 0.05; ** *P* < 0.01; *** *P* < 0.001; **** *P* < 0.0001).

*Sb* dose-dependently suppressed the spontaneous contraction of the CM/LM strips containing all layers. The average contractile amplitude in the CM strips (Fig. 4C) was 0.92 ± 0.22 g before *Sb* was added, but we found it reduced to 0.57 ± 0.10 g (*p* = 0.2517 VS Control), 0.45 ± 0.06 g (*p* = 0.0840 VS Control), and 0.32 ± 0.06 g (*p* < 0.05 VS Control) in the presence of *Sb* 5, 10, and 15 mg, respectively. Likewise, prior to supplementation of *Sb*, the average amplitude of LM strips (Fig. 4D) was 0.51 ± 0.05 g. After the addition of *Sb* 2.5, 5, and 7.5 mg, it was dropped to 0.42 ± 0.04 g (*p* = 0.3242 VS Control), 0.26 ± 0.03 g (*p* < 0.001 VS Control), and 0.17 ± 0.03 g (*p* < 0.0001 VS Control), respectively.

### Immunohistochemical staining for TLR4

**Figure 5:**
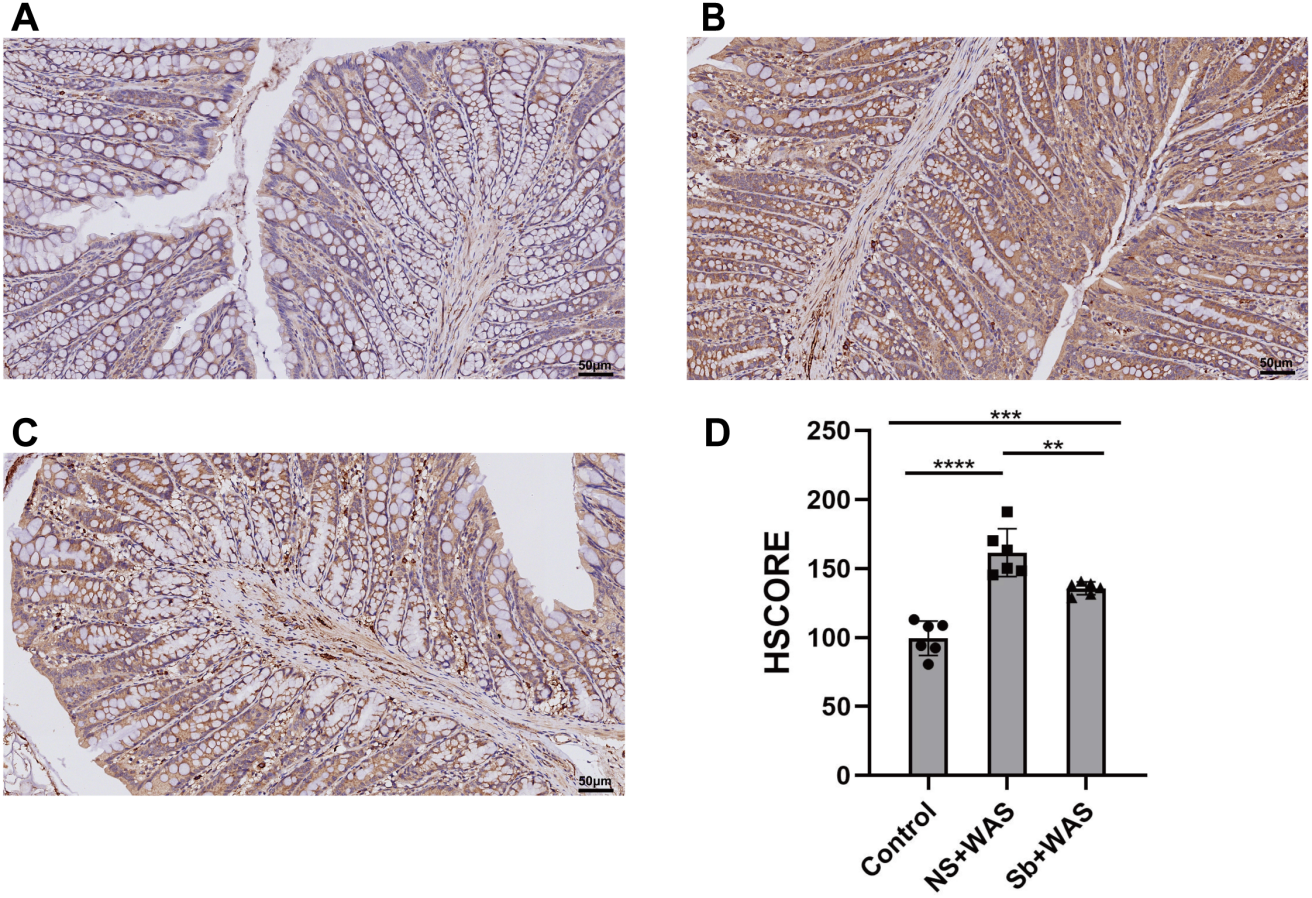
Effects of repeated WAS or *Sb* on the expression of TLR4 in the colon. (A–C) Representative images of TLR4 immunohistochemical staining in three groups of colonic tissues. (D) HSCORE analysis for immunohistochemistry of TLR4. (*n* = 6 per group. ** *P* < 0.01, *** *P* < 0.001).

As shown in Fig. 5, repeated WAS caused a significant upregulation of TLR4 expression levels in rat colonic tissues compared to the control group (*p* < 0.0001). Compared with NS+WAS, TLR4 expression levels in rats in the *Sb* + WAS group were visibly decreased (*p* < 0.01), but still higher than rats in the control group (*p* < 0.001).

### Colon TLR4 mRNA expression

**Figure 6:**
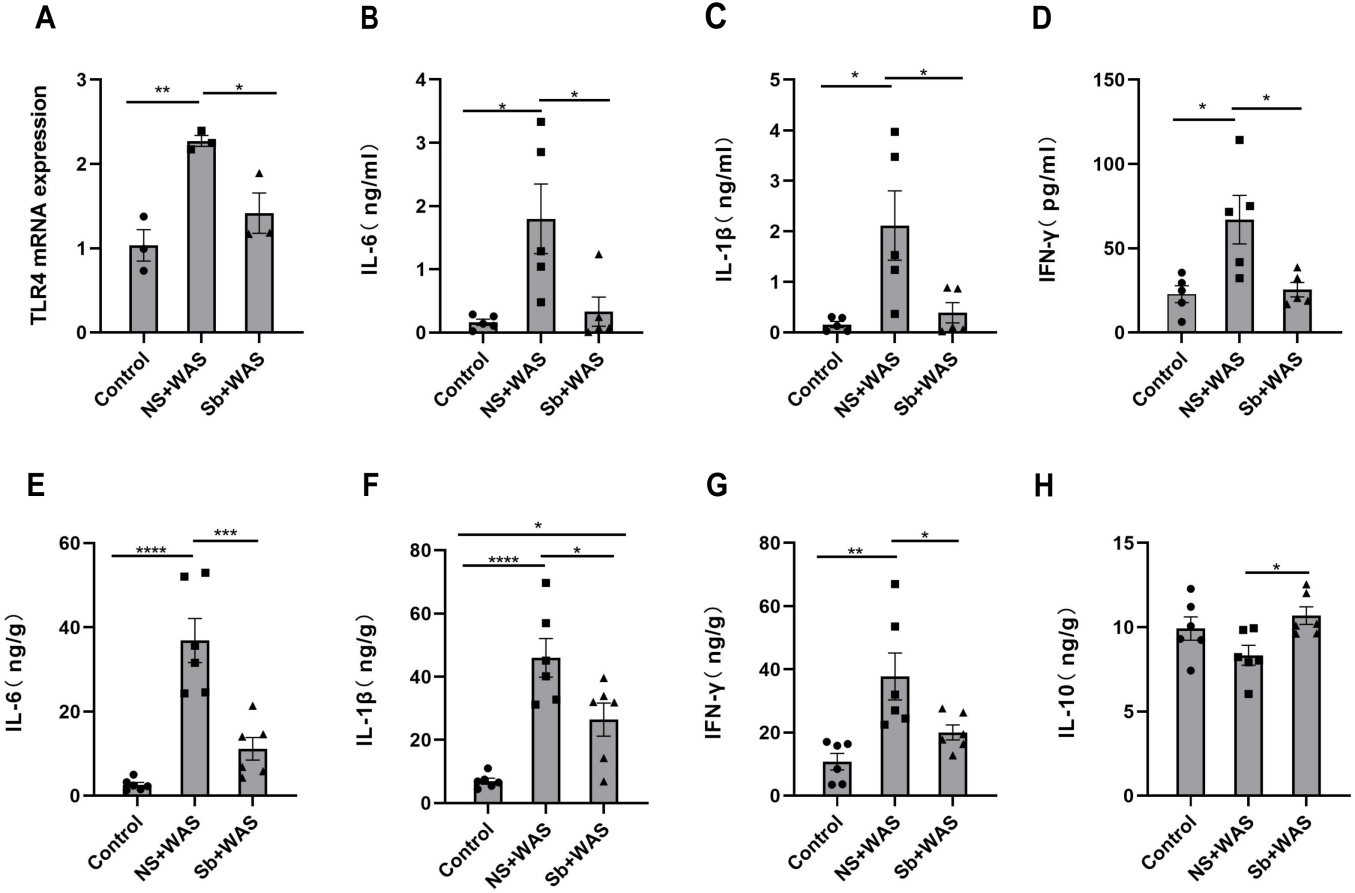
Effects of repeated WAS or *Sb* on TLR4 expression levels in rat colon tissue, and cytokine levels in serum and colonic tissue. (A) TLR4 mRNA expression in colon tissue of three groups of rats. (B–D) Cytokine levels in the serum of three groups of rats. (E–H) Cytokine levels in the colon tissue of three groups of rats. (*n* = 3 − 6 per group. * *P* < 0.05, ** *P* < 0.01, *** *P* < 0.001).

Figure 6A illustrates that the TLR4 mRNA expression level in the NS + WAS group was higher than that in the control group (*P* < 0.01). However, oral intake of *Sb* reversed the higher expression level of TLR4 mRNA due to repeated WAS (*P* < 0.05). The control and *Sb* + WAS groups did not have a significant difference.

### The concentrations of cytokines

The serum IL-6, IL-1β, and IFN-γ concentrations were significantly higher in the NS+WAS group than in the control group (*P* < 0.05), as shown in Figs. 6B–6D. Notably, oral *Sb* treatment significantly reversed the elevation of serum IL-6, IL-1β, and IFN-γ caused by repeated WAS (*P* < 0.05). But IL-10 was not detected in the serum. Meanwhile, the changes in IL-6, IL-1β, and IFN-γ levels in colonic tissue were consistent with those in serum (Figs. 6E–6G). In addition, oral administration of *Sb* upregulated IL-10 levels in the colon tissue of chronic stress-loaded rats (*P* < 0.05, Fig. 6H).

### The gut microbiota diversity of rats

**Figure 7:**
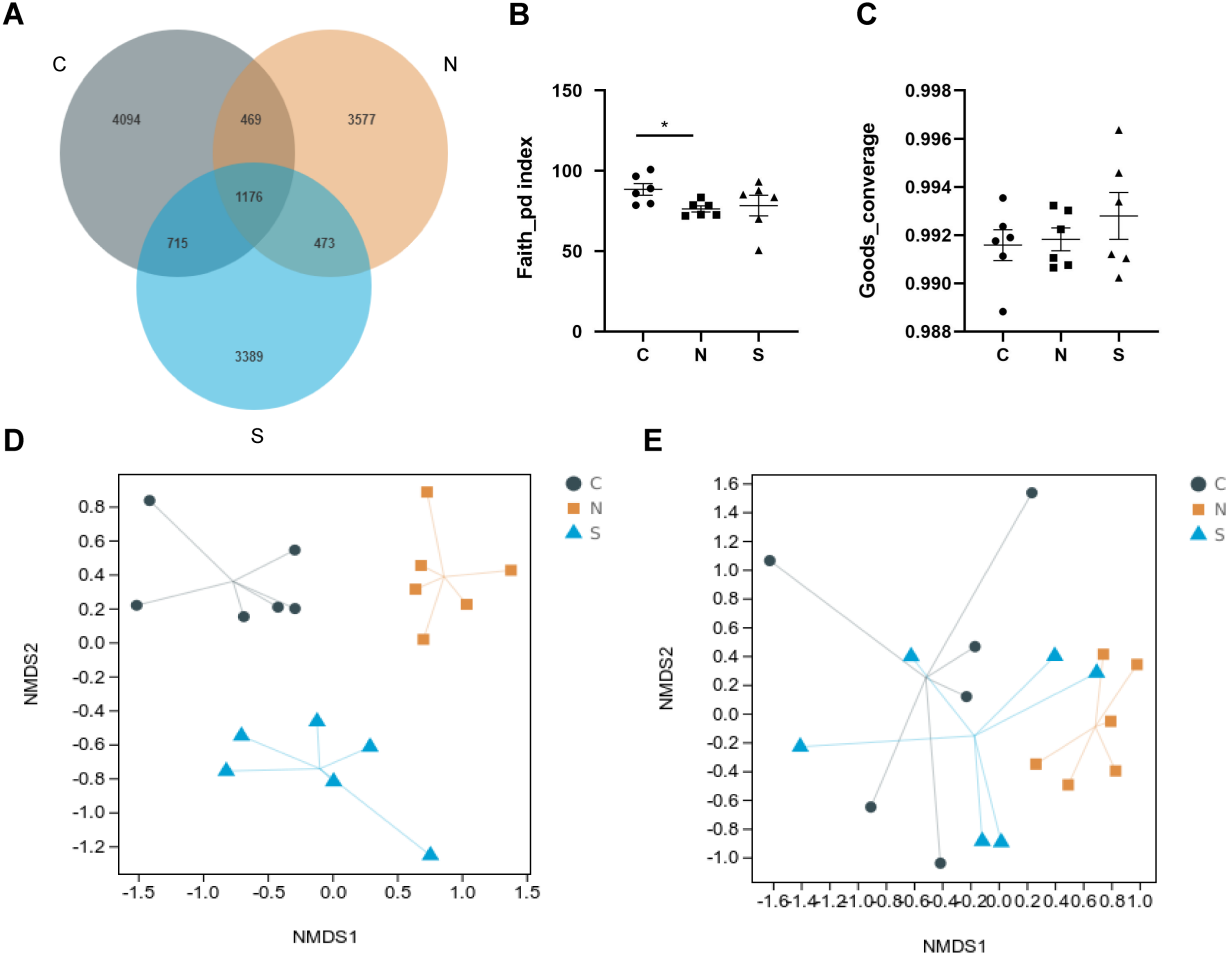
Effects of repeated WAS and/or *Sb* on microbial diversity in in rat feces. (A) Venn diagram of the composition of the microbiota OTU. (B, C) The alpha diversity estimated by the Faith_pd and Goods_converage indexes. (D, E) The beta diversity assessed by unweighted unifrac (*p* = 0.001) and weighted unifrac (*p* = 0.005). (*n* = 6 per group. * *P* < 0.05. C, control group; N, NS +WAS group; and S, Sb +WAS group).

A total of 1,904,405 raw reads were produced from 18 rat fecal samples using high-throughput sequencing. On the basis of 97% sequence similarity, the V3 region and V4 region sequences were identified as 13,893 bacterial OTUs in the rat gut, with 1,176 common bacterial OTUs (Fig. 7A).

As shown in Figs. 7B–7C, repeated WAS decreased the Faith_pd index (*p* < 0.05) in comparison to controls. Furthermore, the results of the Faith_pd index and Goods_converage index indicated a trend towards higher alpha diversity in rats in the *Sb* + WAS group compared to the NS+WAS group, although statistical significance was not reached (Faith_pd, *P* = 0.34; Goods_converage, *P* = 0.42). Meanwhile, NMDS analysis was performed to visualize the difference between individual treatment groups. Three sets of samples were separated and divided into different groups, indicating that beta diversity was significantly different among the three groups (Figs. 7D–7E). These findings indicated dysbiosis in the gut microbiome of rats in the NS+WAS group.

### The gut microbiota composition of rats

**Figure 8:**
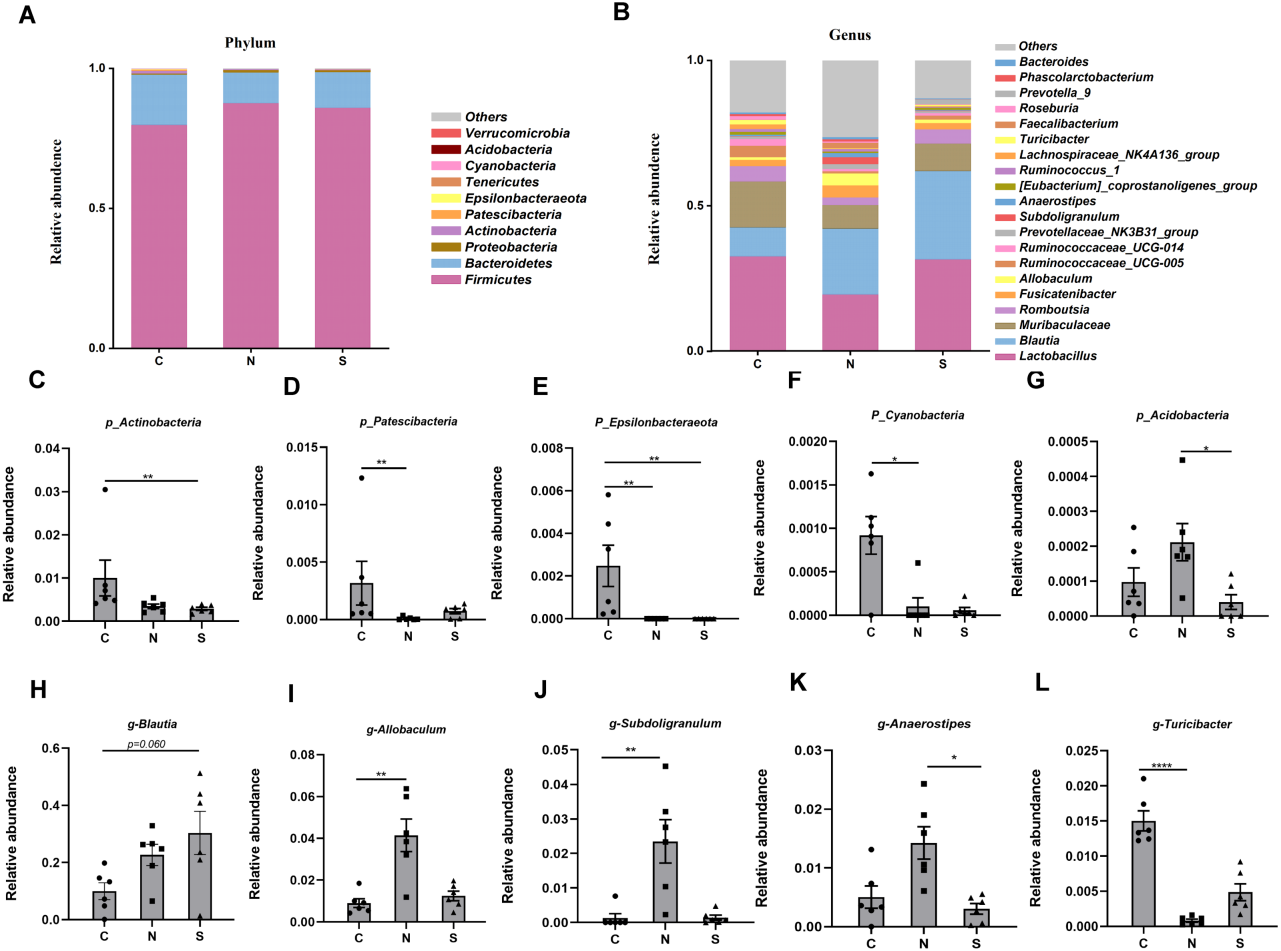
Gut microbiota community composition. (A–B) Fecal microbiota abundance of the top ten phyla and top twenty genera in rats. (C–L) Differences in the relative abundance of microbes among the three groups. (*n* = 6 per group. * *P* < 0.05; ** *P* < 0.01; **** *P* < 0.0001. C, control group; N, NS +WAS group; and S, Sb +WAS group).

At several levels, we compared the differences in microbiota between the three groups. Four phyla were found to have relatively high abundance at the phylum level, with Firmicutes, Bacteroidetes, Proteobacteria, and Actinobacteria being the dominant phyla, which accounted for 99.6% of all bacteria (Fig. 8A).

The NS +WAS group had a greater relative abundance of Acidobacteria than the *Sb* + WAS group (*P* < 0.05, Fig. 8G). When compared to the NS + WAS group, the relative abundances of Patescibacteria (*P* < 0.01, Fig. 8D) and Cyanobacteria (*P* < 0.05, Fig. 8F) were higher in the control group. The control group had a substantially higher relative abundance of Epsilonbacteraeota than the NS + WAS and *Sb* + WAS groups (*P* < 0.01, Fig. 8E). Similarly, the control group had a higher relative abundance of Actinobacteria than the *Sb* + WAS group (*P* < 0.01, Fig. 8C).

Figure 8B shows the top 20 microbial genera. The relative abundances of Allobaculum (*P* < 0.01, Fig. 8I) and Subdoligranulum (*P* < 0.01, Fig. 8J) were lower in the control group, as well as Anaerostipes (*P* < 0.05, Fig. 8K) in the *Sb* +WAS group, when compared to the NS +WAS group. Furthermore, when compared to the controls, repeated WAS reduced Turicibacter relative abundances (*P* < 0.0001, Fig. 8L). Yet, *Sb* supplementation had a tendency to increase the relative abundance of Blautia when compared to controls (*P* = 0.060, Fig. 8H).

**Figure 9:**
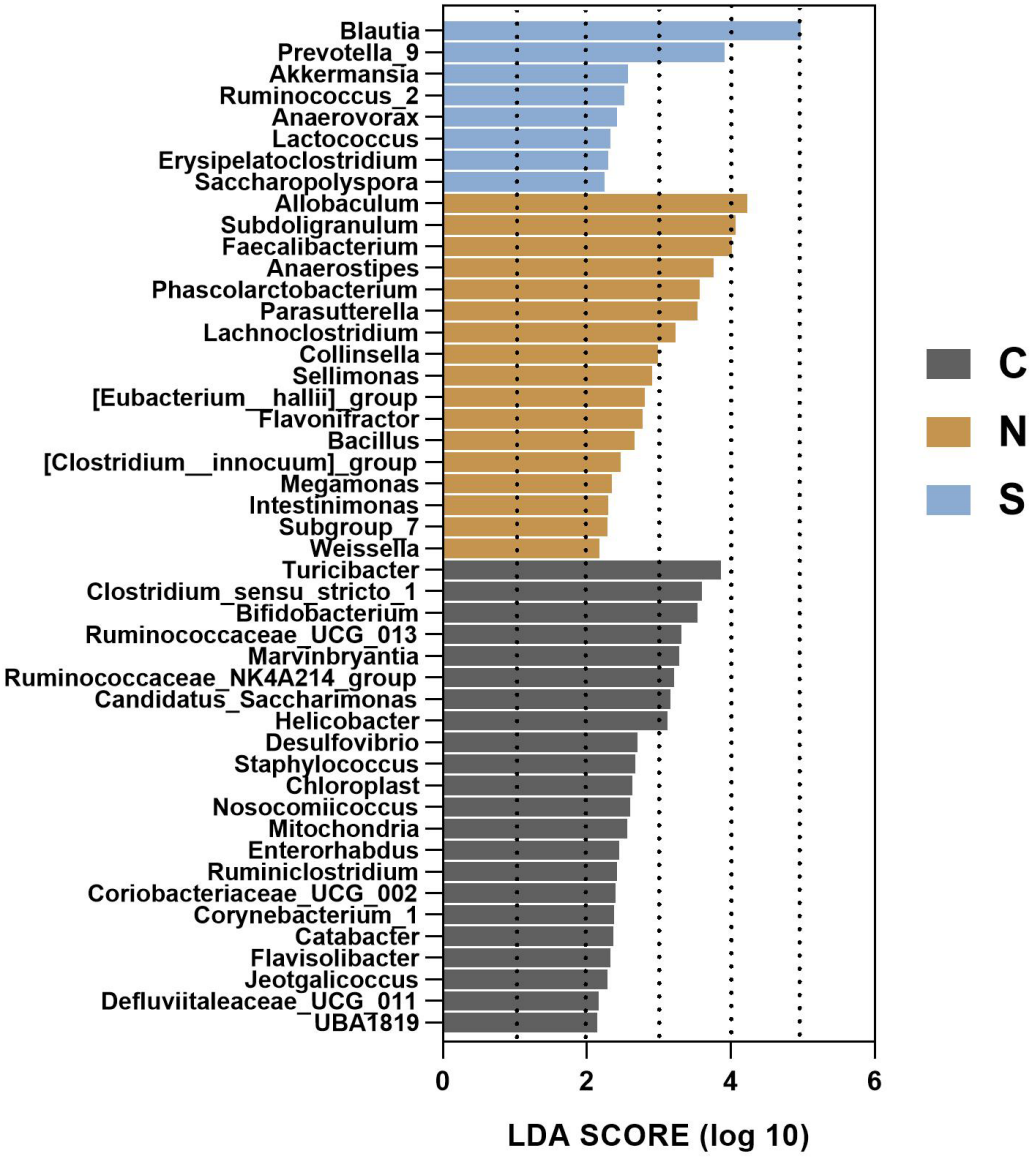
LEfSe analysis of gut microbiota composition among the three groups. (*n* = 6 per group. C, control group; N, NS + WAS group and S, *Sb* + WAS group).

To gain insight into the differences in the gut microbiota communities of the three groups, we performed linear discriminant analysis effect size (LEfSe) analysis at the microbial genus level with LDA scores greater than or equal to 2.0. The control group contained large amounts of Turicibacter, Clostridium_sensu_stricto_1, and Bifidobacterium. The NS +WAS group had higher levels of Allobaculum, Subdoligranulum, and Faecalibacteriumin. A significant enrichment of Blautia was noticed in the Sb +WAS group (Fig. 9).

## Discussion

IBS is a functional gastrointestinal condition that has a substantial influence on patients’ lives and consumes significant healthcare resources (Black & Ford, 2020). IBS is characterized by visceral hypersensitivity and dysmotility (Lin & Yu, 2018). Intestinal inflammation, dysbiosis of the intestinal microbiota, and psychosocial variables may all be involved in the onset of IBS (Ng et al., 2018; Pittayanon et al., 2019; Hauser, Pletikosic & Tkalcic, 2014). There has been substantial evidence of intestinal hypermotility and accelerated transit in IBS-D patients and animal models (Galligan, 2004; Samak et al., 2014; Sweetser et al., 2009). Hypercontractility and hypersensitivity of the intestine are associated with the pathophysiology of IBS-D (Tack et al., 2017). According to previous research, abnormal contraction of intestinal smooth muscle was a possible significant cause of the main symptoms of IBS-D  (Lu et al., 2000). Therefore, reducing gut hypermotility has positive significance for the treatment of IBS-D. Here, animal models of chronic stress-induced IBS-D have been established with repeated exposure to WAS. Based on our experimental data, rats in the NS + WAS group showed increased fecal pellet excretion and enhanced intestinal motility when compared with controls. These results were consistent with the characteristics of diarrhea-predominant IBS. Furthermore, the H&E staining of colonic tissue displayed very little pathology. The above results indicate that the repeated WAS-induced model was successfully generated.

*Sb* is a non-pathogenic probiotic yeast with immune, anti-inflammatory, anti-toxin, and nutritional activity (Pothoulakis, 2009). It is frequently utilized in the treatment of digestive system disorders such as *antibiotic*-associated *diarrhea*. Previous studies have indicated that *Sb* modulates anxiety-related bacteria and increases production of potentially anxiolytic indole metabolites. As a result, anxiety symptoms were improved in a mouse model of fecal transplantation of IBS (Constante et al., 2021). Other studies have also shown that *Sb* improves the quality of life of patients with IBS (Choi et al., 2011). However, many details about the effect of *Sb* on chronic stress-induced colonic hypermotility disease remain unclear. Therefore, we investigated (i) the possible therapeutic effect of *Sb* on colonic hypermotility. (ii) the role of *Sb* in the expression of TLR4 in the rat colon. (iii) the effect of *Sb* on the gut microbiota using 16S rRNA Gene Sequencing techniques.

TLRs are vital for maintaining the balance between microbes and hosts and contribute to the regulation of innate and adaptive immunity. Increased TLR4 signaling in the intestinal epithelium induces alterations in the colonic microbiota (Dheer et al., 2016). A previous study showed that repeated WAS causes visceral pain and increases colonic permeability through TLR4 and proinflammatory cytokine pathways (Arie et al., 2019). Experiments showed that TLR4 expression was increased in the colonic tissue of neonatal maternal deprivation (NMD) induced IBS-like model rats (Mbodji et al., 2011). In addition, TLR2 and TLR4 expression were also found to be higher in IBS-M patients in clinical studies (Belmonte et al., 2012). It can be found that TLRs play a part in IBS. It has been suggested that *Sb* suppresses the intestinal inflammatory response by modulating TLR2 and TLR4, and reducing the production of NF-*κ*B and pro-inflammatory cytokines (Justino et al., 2020). Our preliminary studies demonstrated that repeated WAS up-regulated TLR4 mRNA and protein expression in the rat colon, which fits with previous studies. In the meantime, oral *Sb* reversed these changes, which might account for the therapeutic effects of *Sb*. Although the exact mechanism of IBS is not well understood, the crucial role of low-grade inflammation in the occurrence and progression of IBS is becoming apparent. Recent studies have shown that *Sb* could down-regulate the levels of the proinflammatory cytokines in the blood and tissues of IBS patients. In our study, repeated WAS resulted in significantly higher levels of IL-6, IL-I β, and IFN-γ in rat serum and colonic tissues. The activation of TLR4 results in the release of pro-inflammatory mediators (Cardinal Von Widdern, Hohmann & Dehghani, 2020). We reasonably speculate that elevated inflammatory factors are associated with the upregulation of colonic TLR4. In contrast, oral *Sb* reversed the increase in serum and colonic pro-inflammatory factors induced by repeated WAS and upregulated IL-10 levels in colonic tissues. It has been demonstrated that IL-10 inhibits TLR4-induced inflammation  (Alexander et al., 2021). This explains, to some extent, the anti-inflammatory effect of *Sb*. One study noted that ROS may lead to the upregulation of TLR4 (Hauser, Pletikosic & Tkalcic, 2014). We thus inferred that *Sb* may also downregulate TLR4 expression by suppressing oxidative stress in the colon. Studies have shown that cytokines are involved in the control of gastrointestinal (GI) motility and visceral sensitivity (Hua et al., 2013; Bashashati et al., 2012; O’Malley, Dinan & Cryan, 2011). Therefore, the therapeutic effect of *Sb* on IBS-D may be achieved by modulating the cytokine profiles.

The gut microbiome is sensitive to both stress and therapy, and a consensus has been reached about the value of the gut microbiota to host health (Konturek, Brzozowski & Konturek, 2011). There is considerable evidence of a strong relationship between the gut microbiota and IBS (Martin et al., 2018). In several rodent models, *Sb* administration has induced changes in intestinal flora (Briand et al., 2019; Roy Sarkar et al., 2021; Wang et al., 2019). However, we currently do not know the exact relationship between *Sb*-mediated changes in the intestinal flora and their beneficial effects on chronic stress-induced IBS-D. In the current investigation, repeated WAS decreased the Faith_pd index, while *Sb* supplementation had a tendency to increase the Faith_pd index and Goods_converage index when compared with the NS + WAS group. Similar to a previous study, statistical significance was not reached, which may be related to the small number of animals  (Tao et al., 2021). Alternatively, beta diversity analysis showed that there were significant differences between the three groups. Clinical research showed that the abundance of Actinobacteria was lower in the fecal microbiota of IBS patients compared to healthy people (Rajilic-Stojanovic et al., 2011). The results of our study are consistent with this. One study revealed that *Sb* dramatically increased the abundance of Cyanobacteria and decreased the Firmicutes/Bacteriodetes (F/B) ratio in mice with experimental colitis (Rodriguez-Nogales et al., 2018). However, such changes do not appear in our results, and different doses of *Sb*, different animal species, and different experimental environments may be the reasons for different experimental results. It is worth mentioning that the LEfSe analysis revealed that Blautia had a notable enrichment in the *Sb* + WAS group. The genus Blautia belongs to the Lachnospiraceae family. Since its establishment, Blautia has sparked widespread attention for its function in the treatment of inflammatory and metabolic diseases, as well as its antibacterial action against particular microorganisms (Liu et al., 2021). Cross-feeding of the anaerobic bacterium Blautia with other bacteria facilitates metabolic regulation to a certain extent (Belenguer et al., 2006). Studies have shown that Blautia upregulates intestinal regulatory T cells and produces short-chain fatty acids, contributing to the maintenance of intestinal environmental balance and the suppression of inflammation (Kim, Park & Kim, 2014). In fact, we also found a number of bacteria whose abundance was significantly altered after *Sb administration*. We still know very little about the functions of many of them, but the possibility that changes in the abundance of specific bacteria are involved in the beneficial effects of *Sb* on IBS-D should not be ignored. Yeast could produce ethanol, which has a direct bacterial inhibitory effect. In addition, it has been shown that *Sb* can produce high levels of acetic acid, which has a powerful antibacterial effect (Offei et al., 2019). Thus, intestinal metabolites of *Sb* may be one of the keys to treating stress-induced IBS-D, and it is *worth* digging into. Despite the fact that *Sb* may be advantageous in repeated WAS-induced colonic hypermotility, we still lack insights into the association among the three parameters (colonic motor function, TLR4 and gut microbiome). More research is required to investigate the interrelationships. According to new research, intestinal bacteria may have an essential function in maintaining brain-gut axis health (Mayer, Savidge & Shulman, 2014). Thus, gut microbiota modulated by *Sb* intervention could be a potential treatment option for stress-induced IBS-D.

Our research provided an in-depth analysis of the changes in colonic TLR4 expression and alterations in the gut microbiota that occurred following *Sb* supplementation. In this study, oral *Sb* could alleviate chronic stress-induced colonic dysmotility, regulate the gut microbiota in rats, and reverse higher expression of TLR4 in the colon caused by repeated WAS, indicating a potential therapeutic role for *Sb*. There are still certain limitations to our study. The mechanism of regulation at the cellular level was not probed. In addition, our experiments did not address the source of the inflammatory factors responsible for the visceral changes. More research is required to confirm the exact relationship between stress-induced dysmotility and *Sb*.

## Conclusions

In conclusion, *Sb* improved repeated WAS-induced colonic hypermotility, decreased TLR4 expression in the colon, modulated the cytokines in serum and colon, and regulated the composition of the gut microbiota. Thus, *Sb* may help in the *treatment* of IBS-D.

##  Supplemental Information

10.7717/peerj.14390/supp-1 Supplemental Information 1 Author Checklist

10.7717/peerj.14390/supp-2 Supplemental Information 2 Negative control Magnification is 200 ×

10.7717/peerj.14390/supp-3 Supplemental Information 3 Raw data: Colonic tissue

10.7717/peerj.14390/supp-4 Data S4 Experiment-related raw data

## Additional Information and Declarations

### DNA Deposition

The following information was supplied regarding the deposition of DNA sequences: NCBI:BioProject PRJNA813982

https://www.ncbi.nlm.nih.gov/bioproject/PRJNA813982.

### Data Availability

The following information was supplied regarding data availability: The raw data is available in the Supplemental Files.
