# Supplementary figures and images for: The effects of Saccharomyces boulardii on rat colonic hypermotility induced by repeated water avoidance stress and the potential mechanism
Source: PeerJ. 2022 Nov 22;10:e14390. doi: 10.7717/peerj.14390 (PMC9695494; doi:10.7717/peerj.14390)

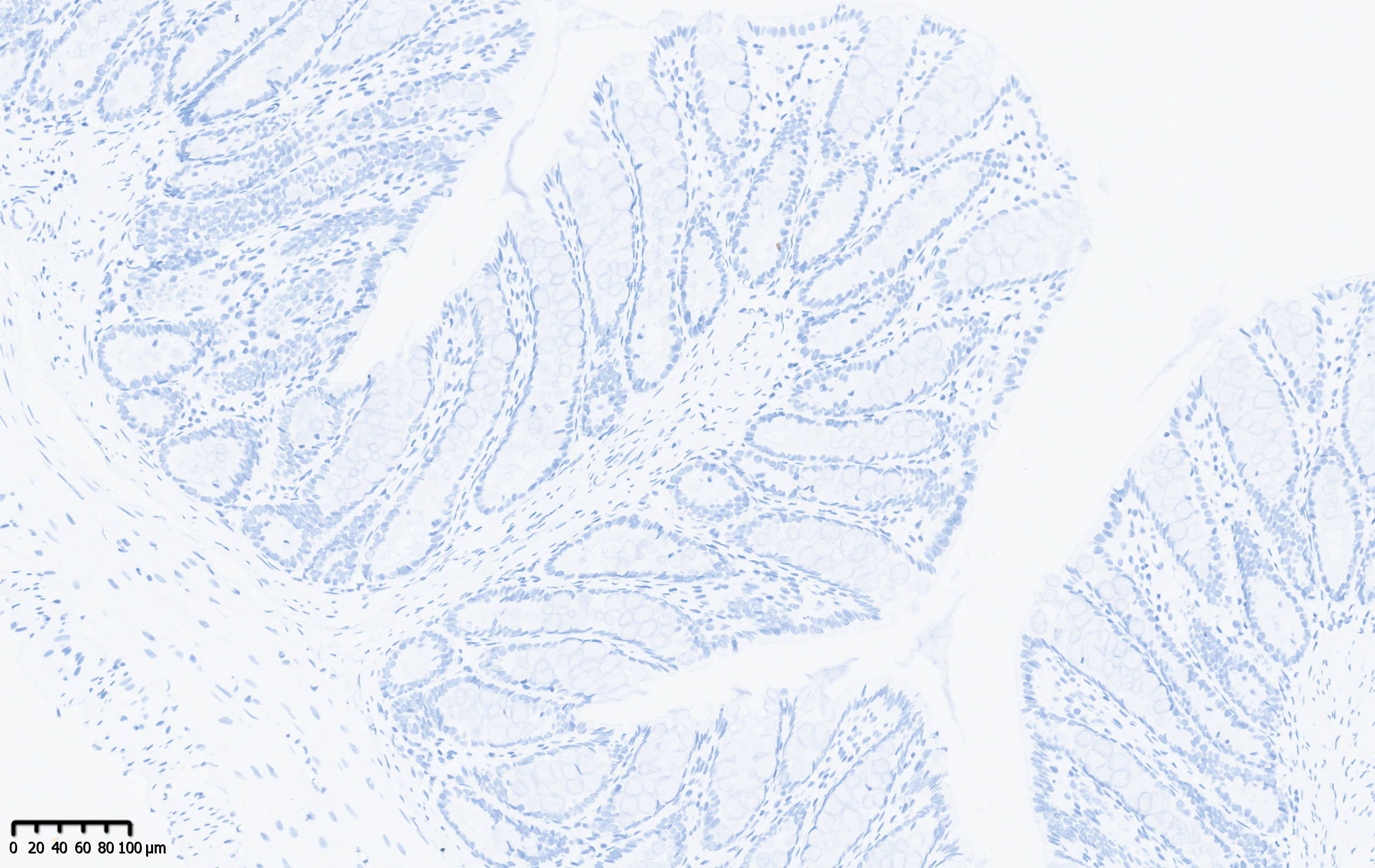

Supplement: Supplemental Information 2 — Magnification is 200 × [file peerj-10-14390-s002.png]
